# Another Best Practice: Leveraging User and Stakeholder Perspectives to Improve and Refine Existing Medical Products

**DOI:** 10.9745/GHSP-D-23-00494

**Published:** 2023-12-22

**Authors:** Kevin J. Peine, Madeleine Short Fabic, Stephen Hodgins

**Affiliations:** a Office of Population and Reproductive Health, U.S. Agency for International Development, Washington, DC, USA.; b Associate Editor, Global Health Science and Practice.; c Editor-in-Chief, Global Health Science and Practice; School of Public Health, University of Alberta, Edmonton, Canada.

## Abstract

Refinement of existing medical products, which may already have an established evidence base, robust market, and experienced users, may better meet user and potential user needs, if feedback from key stakeholders is solicited and incorporated early in the refinement process.

See related articles by Burke et al. and Callahan et al.

## INTRODUCTION

With more than 74 million users, injectable contraceptives are one of the most widely used methods of contraception globally and remain the most prevalent method in sub-Saharan Africa.^1^ Depo medroxyprogesterone acetate (DMPA) is the most commonly used injectable contraceptive and is available in both a 3-month intramuscular formulation (DMPA-IM, 150 mg/ml) administered by providers and a 3-month subcutaneous formulation (DMPA-SC, 104 mg/0.65 ml), which is preloaded into a Uniject and can be either administered by providers or self-administered. Although both DMPA formulations are widely used, discontinuation is common; clients frequently cite concerns about side effects (e.g., contraceptive-induced menstrual changes) and delays in expected return to fertility.^2^^,^^3^ Adequate counseling can ease such concerns.^4^ However, for clients experiencing negative side effects, counseling cannot alleviate the symptoms. These clients have the unenviable choice of either continuing despite distressing side effects, switching to another method that is possibly less effective, or discontinuing and facing the risk of unintended pregnancy.

To meet the family planning needs of these individuals and others who do not want to become pregnant but have concerns about hormonal method use, it is critical to facilitate access to a wider range of methods, including through the development of new nonhormonal contraceptive options. Recognizing that research and development of promising new products will take time, it is also important to refine existing products to increase accessibility, affordability, acceptability, and satisfaction. Although studies that yield incremental improvements to existing contraceptive methods will not address every method-related concern, this research may reduce some of the barriers users face to access and continue using their chosen method. This work can include studies to:

- **Extend the duration of effectiveness of an existing product**: Prior work demonstrated the 3-year etonogestrel implant is safe and effective for 5 years.^5^ Additionally, research has resulted in a label extension of the 52 mg levonorgestrel intrauterine system (IUS) from up to 5 years to 8 years (Mirena),^6^ while the effectiveness of another 52 mg levonorgestrel IUS (Liletta) has recently been assessed up to 8 years.^7^ This work has the potential to reduce unnecessary cost and inconvenience of method removal and reinsertion for those who wish to continue their chosen method.
- **Reduce side effects of existing products**: Prior work has aimed to improve user experience of existing methods through concurrent use of other existing low-cost interventions. For example, studies have shown that the use of nonsteroidal anti-inflammatory drugs alongside intrauterine device insertion can reduce pain and heavy bleeding, both of which are common method-related concerns.^8^
- **Establish how existing methods may influence users' overall health**: A recently launched clinical trial in Kenya seeks to determine whether reduced menstrual blood loss from the use of levonorgestrel IUS can alleviate iron-deficiency anemia (NCT05233956). Some side effects of progestin-based contraceptive methods, including contraceptive-induced menstrual changes, are largely unavoidable; however, research may also provide insights on potential noncontraceptive health benefits of certain products, which may improve informed choice and enable users to choose a method that better fits their overall health needs.

Such approaches help to ensure users' needs and preferences are better met by the existing method mix. However, introduction and scale-up of a refined method may face a different set of challenges than typically seen with a first-in-class or novel product. Unlike the processes for new products, which employ mostly well-established regulatory pathways and attempt to create awareness, understanding, and demand, refinements of existing products may face different regulatory considerations, have an existing market with established knowledge and opinions of the product, and require updated sets of guidelines for counseling clients and training providers on a product with which they may already have experience.

With this refinement approach to better meeting client needs in mind, we want to draw attention to the promising results of 2 complementary articles by Burke et al.^9^ and Callahan et al.^10^ published in this issue of *GHSP*. Both articles offer programmatic lessons that can be applied if and when DMPA-SC duration-of-effectiveness guidelines shift. While the lessons from these articles are specific to DMPA-SC, we note that the questions raised and the stakeholders included mirror those that would be of critical importance when pursuing refinements to any existing medical technology. As demonstrated in these articles, soliciting feedback from policymakers, providers, users, and their partners should be done early in the research and development process. This type of work can generate useful evidence to inform product developers on priority refinements (e.g., preferred duration of effectiveness) and possible regulatory routes that could be pursued (e.g., policymakers' preference for label change versus normative guidance updates).

Shifts in DMPA-SC duration-of-effectiveness guidelines would present unique opportunities to expand the contraceptive method mix to better meet individuals' and couples' contraceptive needs. We believe it is imperative to begin the planning process for such a shift now and commend the Burke and Callahan study teams for providing useful evidence to inform planning.

Questions raised by articles by Burke and Callahan are relevant when pursuing refinements to any existing medical technology.

## NOTABLE FINDINGS, INCLUDING POTENTIAL BENEFITS, OF REFINING AN EXISTING METHOD

Burke et al.^9^ and Callahan et al.^10^ report results from 2 studies conducted in Lagos, Nigeria and Kampala, Uganda, eliciting feedback from current and potential injectable contraceptive users, partners of current and potential contraceptive users, as well as providers, policymakers, and other key stakeholders, on potential 4- and 6-month duration DMPA-SC products. Both studies build on 2 recent clinical studies, 1 of which found that the existing (3-month) DMPA-SC product is safe and reliably effective for 4 months^11^ (or potentially longer) and the other of which is exploring the efficacy of a 6-month DMPA-SC product. To facilitate the potential introduction of these DMPA-SC duration-of-effectiveness updates, Callahan et al.^10^ conducted 48 in-depth interviews and 11 focus group discussions with 92 service providers and other stakeholders involved in service delivery, program management, and policymaking. Burke et al.^9^ conducted 33 focus group discussions with 118 participants and 2 ideation workshops with 47 participants, who were either current injectable users, potential injectable users, or men who had not undergone vasectomy and were in a sexually active relationship with a woman. Both studies found resoundingly positive views concerning 4-month and 6-month dose frequency with some important nuances (Table).

**TABLE.** Perceived Benefits and Challenges of Potentially Adding 4-Month and/or 6-Month DMPA-SC Into the Method Mix

	Callahan et al.^10^ Providers/Managers	Burke et al.^9^ Users/Potential Users
Perceived benefits would be to:	Give women more choices to better meet their needs Increase client satisfaction Reduce the frequency of clinic visits Reduce travel time and costs to users	
	Expand the pool of users Reduce supply chain costs to governments, including through potentially simplified product procurement logistics and storage Reduce the burden on providers	Increase privacy due to fewer facility visits Make reinjection window and appointments easier to remember Potentially reduce the time to return to fertility Potentially reduce side effects Increase shared decision-making and couple communication
Perceived challenges would be to:	Possibly pay a higher cost Navigate a narrower reinjection window and/or longer time between doses (e.g., client trouble returning on time; less flexibility to manage schedules) Reduce provider/client interaction opportunities Understand and navigate whether/how the different duration-of-effectiveness windows impact side effects	
	Create potential confusion among clients (e.g., differentiating between methods) Maintain sufficient stock Navigate client counseling on the new method Add extra reporting burden	Potentially delay (even further) return to fertility Potentially increase duration/severity of side effects

As previously discussed, while DMPA is widely used, concerns about side effects, including contraceptive-induced menstrual changes and delayed return to fertility, are prevalent among users, potential users, and their partners.^2^^,^^3^ One potential pathway to reducing or alleviating some of these effects is through a reduction in dose exposure among users—either by reducing the amount of hormone in each dose or by reducing the dosing frequency.^12^ This hypothesis was evaluated during a recent clinical study comparing effectiveness of doses of 45 mg, 75 mg, and 105 mg of the DMPA-IM formulation delivered subcutaneously to the currently marketed 3-month DMPA-SC product at a dosage of 104 mg.^13^ While the study found that doses lower than the current DMPA-SC dose did not suppress ovulation sufficiently to pursue a lower dose product, the 105 mg subcutaneous injection did reliably suppress ovulation for a 4-month period. This finding adds evidence to the study (described earlier) demonstrating that the existing (3-month) DMPA-SC product is safe and effective for 4 months.^11^ These findings, suggesting that the currently marketed DMPA-SC product is likely effective for a longer period than its 3-month label, provide the evidence enabling important changes for DMPA-SC service delivery, including either a change in dosing frequency or a change in the length of the reinjection window.

The first change could be a label change or authorization of off-label use of a 4-month product, which would reduce the number of doses required per client by 25%. This reduction in dose frequency plausibly could lead to overall lower side effect profiles and a more predictable return to fertility, though evidence of these potential benefits is still needed. In addition, in the context of financial constraints—for clients, ministries of health and finance, and donors—such a label change could lead to an overall cost reduction per user, thereby lowering costs incurred by both clients and procurement agencies. A reduction in dose frequency could also benefit overburdened health systems by decreasing the number of visits required to family planning providers and reducing the volume of sharps waste generated and disposed of. Both Burke et al.^9^ and Callahan et al.^10^ reference work on a potential 6-month DMPA product that, if demonstrated effective, could reduce procurement costs and provider visits by up to 50%. In fact, Callahan et al.^10^ noted that a key consideration for policymakers in Uganda was the potential benefits to local supply chains of reduced procurement volume.

Even without a label change, the reinjection guidance could be revised. Specifically, recognizing that DMPA-SC suppresses ovulation for at least 4 months, the reinjection guidance could be updated to allow for a longer reinjection window. Doing so would not yield the 25% dose reduction previously highlighted. However, a common reason for discontinuation of DMPA is missing the allowable window for reinjection (i.e., 2 weeks, up to 15 weeks from the last injection)^14^ and having to restart the product. Extending this window to 4 (or more) weeks supports users to receive a reinjection at a time that works best with their lives, minimizes the impact that brief stock-outs have on users seeking reinjection, and helps ensure that individuals are not facing unnecessary barriers to accessing their chosen method. The Callahan et al.^10^ study found that some experts were most comfortable with extending the reinjection window, rather than changing dosing frequency, because the 4-month duration product may result in an even shorter window for reinjection (i.e., 1 week) than the existing DMPA-SC product.

The information generated by Burke et al.^9^ and Callahan et al.^10^ on the opportunities and drawbacks providers, users, and potential users perceive concerning the potential label or reinjection guidance changes is critical for determining how best to frame market introduction and guideline development for a revised DMPA-SC product. Again, across both studies, study participants of all categories were keen to introduce methods that better meet women's contraceptive needs and recognized the opportunity of these DMPA-SC shifts to do just that.

## WHAT WILL IT TAKE TO IMPLEMENT DMPA-SC DOSING INTERVAL CHANGES?

Key tasks required to implement these potential DMPA-SC duration-of-effectiveness changes include addressing regulatory and policy needs; identifying and addressing provider, user, and potential user concerns; and identifying and addressing ethical questions raised by new evidence on DMPA-SC duration of effectiveness. We highlight several key considerations for each of these issues, though these reflections are not exhaustive.

### Addressing Regulatory and Policy Needs

Modifying the dosing regimen of an existing product may give rise to unique regulatory and policy challenges. Regulatory agencies, such as the U.S. Food and Drug Administration, encourage reliance on exposure-response studies^15^ to determine changes to dosing regimens (e.g., dose or dosing interval) that were not studied in original clinical trials. Evidence from the 2 clinical trials previously described could meet this regulatory requirement. Meanwhile, in the absence of regulatory changes, with encouraging clinical trial evidence, providers and/or ministry of health policymakers could move forward with “unapproved use of an approved drug,” that is, “off-label” use of DMPA-SC. Callahan et al.^10^ included policymakers in their service delivery consideration study and asked whether off-label use may be acceptable and feasible within their country and context. They found that most policymakers would be supportive of such use if evidence on product efficacy and safety were sufficiently robust. Respected global normative bodies like the World Health Organization (WHO) may be well placed to provide science--based and trusted opinions on the strength of evidence and recommendations for off-label use. The WHO's *Selected Practice Recommendations for Contraceptive Use*^16^ noted, “repeat DMPA injection can be given up to 4 weeks late without requiring additional contraceptive protection.” These updated WHO recommendations could offer a pathway to engage with policymakers and guideline developers about making DMPA-SC a 4-month product within their service delivery programs.

### Identifying and Addressing Concerns of Providers, Users, and Potential Users

Although adjusting guidance on the effectiveness duration of a longstanding and widely used product has the benefit of reaching an established market of end users, it may also raise questions among providers, existing users, and potential users. As Burke et al.^9^ and Callahan et al.^10^ found, providers or clients may have concerns that too many similar products and/or too long a duration between doses may create confusion among users and potential users. They may also have concerns about how the product may alter the user experience. With respect to extending the time between injections, there may be a set of interrelated misconceptions to overcome, including that the dosage of hormones is higher with 4- or 6-month DMPA-SC products, the side effects are worse, or that predictable return to fertility is longer. In reality, use of the 4-month and 6-month DMPA-SC products would result in **lower** cumulative drug exposure, which would potentially result in fewer side effects and a more rapid return to fertility (though data testing these theoretical advantages are not yet available).

Thanks to the research conducted by Burke et al.^9^ and Callahan et al.,^10^ program managers and policymakers have key stakeholder perspectives to inform decisions on the introduction of refined versions of DMPA-SC products and guidelines, including a set of processes and new knowledge to help ensure that counseling, messaging, and marketing are relevant to local context. Moreover, much of the data collected provides valuable early market insights that can be used to build on drivers of demand for such products, including information on desired cost and willingness to pay. For example, while off-label use may result in the largest cost reduction for clients and programs, changes to existing labels may result in cost increases to the manufacturer, which may, in turn, be passed on to clients. The article by Burke et al.^9^ and studies of these types provide critical information to assess whether clients may be willing to accept a marginally higher cost for a longer-acting product. Such evidence is not only relevant to potential DMPA-SC changes but is also applicable to broader family planning programs as it helps to identify contraceptive characteristics of importance to current and potential users. This type of information can be used for myriad purposes, including for developing materials that support clients to consider and choose from the full range of options with frequently asked questions that specifically address client (mis)perceptions, including how long or short the time is between method discontinuation and expected return to fertility.

### Addressing Ethical Questions

Given the many benefits that a potential extended-duration DMPA-SC product may yield, it is important also to consider potential ethical issues associated with the products included in the offered method mix. In particular, if increasing evidence shows that injectables are effective in preventing pregnancy for 4 months, 6 months, or even longer, is it ethical to continue to offer injectables at 3-month intervals? This question is especially relevant as current injectable products have unwanted side effects and longer-than-desirable return to fertility. If the dosing is more frequent than required to suppress ovulation, are injectable users being unduly burdened? At a minimum, counseling guidelines must be regularly revisited and revised to reflect insights emerging from scientific advances to help ensure that providers offer quality counseling based on up-to-date information and that clients are well positioned to make informed choices.

## CONCLUSIONS

The Burke et al.^9^ and Callahan et al.^10^ articles serve as strong case studies demonstrating an approach to the development and introduction of refined medical products. While the results are specific to DMPA-SC duration-of-effectiveness guidelines and programming, the research approach of these studies is widely applicable to medical product research advances—from more simplified dosing regimens, as with the human papillomavirus vaccine,^17^ to combining existing individual products into multipurpose prevention technologies.^18^ The global health field has increasingly incorporated human-centered design and stakeholder perspectives into research and development. It is imperative that such approaches are also incorporated into the refinement of existing products. Research on refinement of such products, which may already have an established evidence base, robust market, and experienced users, can answer questions unlike those typically asked for novel products. As the number of products increases within and beyond the family planning field, more opportunities will arise to refine existing technologies, especially in the absence of potentially “game-changing” products that can take decades to come to fruition. Research methods used in studies like those of Burke et al.^9^ and Callahan et al.^10^ help to ensure that end users and communities have a voice in driving global health decisions. We encourage more research of this type. Namely, we encourage those funding and implementing medical product refinement research to concurrently fund and implement behavioral research to identify and address the perspectives of users, potential users, providers, policymakers, and other key stakeholders.
